# Optimization of a novel dental self-healing resin composite by bacteria-induced biomineralization

**DOI:** 10.3389/fbioe.2025.1590348

**Published:** 2025-06-02

**Authors:** Yanyan Han, Xiaoxuan Zhang, Jianing Weng, Shiqi Tian, Xian Dong, Zhiheng Cai, Yi Zhang, Tiantian Wu, Dan Lin, Yaqin Zhu

**Affiliations:** ^1^ Department of General Dentistry, Shanghai Ninth People’s Hospital, Shanghai Jiao Tong University School of Medicine, Shanghai, China; ^2^ College of Stomatology, Shanghai Jiao Tong University, National Center for Stomatology, National Clinical Research Center for Oral Diseases, Shanghai Key Laboratory of Stomatology, Shanghai Research Institute of Stomatology, Shanghai, China; ^3^ Shanghai Jiao Tong University School of Medicine, Shanghai, China; ^4^ Shanghai University of Medicine and Health Sciences, College of of Medical Technology, Shanghai, China

**Keywords:** self-healing, bacteria, biomineralization, MICP, CaCO3

## Abstract

**Introduction:**

Dental resin restorations often fail due to microcrack expansion, causing fractures and secondary caries. Self-healing resin composites based on Microbially Induced Calcium Carbonate Precipitation (MICP) offer a solution. In these composites, moisture and air activate bacteria to precipitate calcium carbonate (CaCO_3_) and repair microcracks. When a crack seals, bacteria become dormant or form spores until the next crack forms, triggering repeated self-healing.

**Methods:**

This study involved the optimization of nutrients to enhance biocompatibility, the preparation of dental resin composites incorporating eight different bacterial strains, the investigation of Mn^2+^ to enhance self-healing properties, and the utilization of a method to evaluate self-healing efficiency tailored for the oral environment. This method took a microscopic view of the healing process in artificial saliva, and the self-healing efficiency was determined by quantifying the scratch area.

**Results:**

In the final results, *Bacillus* sphaericus (ATCC 4525) cultured with Mn^2+^ exhibited the most impressive self-healing effect, while *Bacillus* pasteurii (B80469) had the weakest self-healing effect in the study. Otherwise, Bifidobacterium longum showed no significant difference between its initial and secondary healing effects.

**Discussion:**

This dental self-healing resin composite can undergo multiple rounds of self-repair and boasts high biocompatibility, leading to a significant reduction in the failure rate of dental resin restorations.

## 1 Introduction

Dental caries, the most common oral disease affecting individuals of all ages (Organization, 2022), poses a significant challenge in maintaining oral health. Traditional dental resin composites are widely used for cavity fillings due to their ease of application and aesthetic appeal, however, they have limitations, including being susceptible to microcracking from the pressure of chewing and thermal stress (Deligeorgi et al., 2001), these microscopic cracks are difficult to detect and repair, leading to issues such as damage to the restoration and fractures, and the formation of secondary caries. To address these challenges, current research efforts were focused on improving the inorganic fillers and incorporating reinforcements in composites to prevent cracking (Nitta et al., 2017; Münchow et al., 2018; Wang et al., 2024), however, despite these efforts, resin restorations continue to face challenges with persistent fractures.

As a response to this issue, self-healing dental resin composites have been developed and studied by Wertzberger et al. (2010), which showed promise results in extending the lifespan of dental resin restorations, providing both social and economic benefits. Research on self-healing dental resin composites has mainly focused on utilizing PUF microcapsules for self-repair. When microcracks or damage occur in resin composites, the microcapsules rupture and release a healing agent to repair the cracks. The self-healing performances of this system have been demonstrated to restore between 25% and 80% of the original fracture toughness (Wertzberger et al., 2010; Huyang et al., 2016; Wu et al., 2016; Yue et al., 2018; Menikheim et al., 2022; Yao et al., 2022). While the microcapsule self-healing system has shown promising results in self-repair and crack suppression, it has limited self-healing ability and some degree of biological toxicity.

**FIGURE 1:**
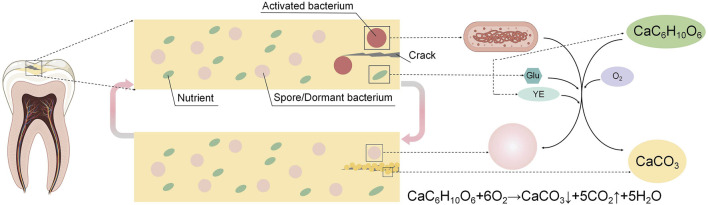
Self-healing mechanism of dental microorganism self-healing resin composites: As a microcrack occurs, moisture and air infiltration trigger the bacteria to produce calcium carbonate (CaCO_3_), facilitating the natural healing process of the microcracks. Once the cracks are sealed, the bacteria become inactive due to the lack of water and oxygen until the microcracks reoccur. At that point, the bacteria are reactivated and restart repairing the microcracks.

Calcium carbonate (CaCO_3_) is a naturally mineral byproduct of microbial metabolism, its application for crack repair of building structure was first proposed by Gollapudi et al. (1995) in 1995, and later expanded upon by Ramachandran et al. (2001) in 2001 with the concept of Microbially Induced Calcium Carbonate Precipitation (MICP) for crack repair in concrete. MICP involves microbial metabolism forming CO_3_^2-^, which combined with Ca^2+^ in the environment, leads to the precipitation of CaCO_3_ crystals for biomineralization. Microbial self-healing concrete, which uses microbes activated by moisture or oxygen to form biogenic CaCO_3_ and fill cracks, reduces permeability and enhances durability, and it is widely used for its effectiveness, sustainability, and low toxicity (Jonkers et al., 2010; Van Tittelboom et al., 2010; Wiktor and Jonkers, 2011; Wang J. et al., 2012; Seifan et al., 2016a; Van Tittelboom et al., 2016), with ongoing research focusing on optimizing factors like bacterial strains, nutrients, and aeration (Okwadha and Li, 2010; Achal et al., 2011; Khaliq and Ehsan, 2016; Xu and Wang, 2018; Xu et al., 2018). The primary process of MICP occurs through the nitrogen cycle, with bacteria capable of producing essential protein enzymes with mineralizing properties through metabolic processes, such as urease, which aids in the formation of CaCO_3,_ such as *Bacillus pasteurella*, *Bacillus* sphaericus, *Bacillus* licheniformis, and *Bacillus subtilis*, playing crucial roles in inducing carbonate precipitation. Seifan et al. (2020) incorporated Generally Recognized As Safe (GRAS) bacteria into dental resin composites to enable self-healing of microcracks through CaCO_3_ formation, activated by moisture and air, with bacteria cycling between active and dormant states for repeated repairs. Their study demonstrated that Bifidobacterium longum and *Bacillus* licheniformis could effectively induce biomineralization in Z250 dental resin composites. However, to address cytotoxicity associated with the nutritional components including urea and calcium chloride utilized in the study, biocompatible calcium lactate and glucose were introduced as alternative nutrients, showing great potential for enhancing dental materials (Figure 1).

This study investigated the first and secondary self-healing abilities of eight different strains of bacteria in dental resin composites by creating microcracks on resin samples and observing the healing progress of these microcracks at different time intervals in a simulated oral environment, to assess accurate self-healing efficiency which was measured quantitatively through crack area measurements, and the strains that demonstrated the highest self-healing efficiency in dental resin composite were identified, providing valuable insights and data for the advancement of self-healing dental resin composites, which aims to reduce the occurrence of fracture and the incidence of secondary caries, thereby extending the service life of the dental resin composites.

## 2 Materials and methods

### 2.1 Strains and materials

Bacterial strains which were GRAS including ATCC4525 *Bacillus* sphaericus (BaSph-A) and ATCC9789 *Bacillus* licheniformis (BaL-B) were acquired from Shanghai Rongmin Biotechnology Center, BMZ008183 Lysinibacillus sphaericus (BaLy), ATCC9859 *Bacillus* licheniformis (BaL-A), B80469 (sourced from NCIM2477) *Bacillus pasteurella* (BaP), B81617 (sourced from ATCC15702), Bifidobacterium longum (BiL), BMZ140479 (sourced from ATCC55739) *Lactobacillus* reuteri (LaR) and LMG22257 *Bacillus* sphaericus (BaSph-B) were obtained from Ningbo Mingzhou Biotechnology Co., Ltd additionally.

Calcium chloride, urea, yeast extract, CASO medium (soy peptone casein digest), De Man, Rogosa and Sharpe Medium (MRS) medium, Brain-Heart Infusion Broth (BHI) and Nutrient Agar (NA) medium were all sourced from Beijing Pufei Biotechnology Co., Ltd. MnSO_4_.H_2_O (99%) was purchased from Macklin Biochemical Technology Co., Ltd. (Shanghai, China). Barium Aluminosilicate Glass (NF180nm, BAS Glass) was produced by Schott AG Co., Ltd (LandShut, Germany). Bisphenol A glycidyl methacrylate (Bis-GMA), tri(ethylene glycol) dimethacrylate (TEGDMA, 95%), camphorquinone (CQ, 97%), ethyl 4-dimethylaminobenzoate (4-EDMAB, 99%) were all purchased from Sigma-Aldrich Reagent (Shanghai, China). The artificial saliva (pH 6.8) was obtained from Phygene Life Sciences Company (Fuzhou, China). Ethanol (75%) was purchased by Dongyi Chemical Co. Ltd (Shanghai, China). Pure water (H_2_O) was prepared by an ultrapure water system (Direct-Pure UP With Dispenser UP 10 UV), purchased from Rephile Corporation (Shanghai, China). All chemicals reagents were utilized without further purification.

### 2.2 Cultivation of strains

The strains were inoculated at a volume fraction of 1‰ into various culture media that had been sterilized using high pressure, then incubated for 48 h in a shaking incubator set at 150 rpm (specific culture conditions in Table 1). After incubation, the culture medium was transferred onto nutrient agar plates containing 15 g/L agar, and spread evenly across the entire surface, then left to cultivate for an additional 48 h, finally the bacteria that grew on the agar plates were used as the initial strains. These initial strains were then inoculated at a volume fraction of 1‰ into the corresponding culture media that had been sterilized by high pressure. They were incubated for 48 h again in a shaking incubator set at 150 rpm, resulting in liquid cultures of either *Bacillus* or non-Bacillus strains. To further boost spore production referring to previous studies (Xu et al., 2018), 10 mg/L of MnSO_4_.H_2_O was added to the final culture medium containing BaSph-A, referred to as BaSph-Mn, in addition to the strains cultured separately.

**TABLE 1:** Cultivation conditions of different strains.

Stain	Stain abbreviation	Serial number	Aerobic style	Temperature (°C)	PH	Culture medium
*Lactobacillus* reuteri	LaR	BMZ140479 (from ATCC55739)	Anaerobe	37	7.0	MRS
Bifidobacterium longum	BiL	B81617 (from ATCC15702)	Anaerobe	37	7.0	BHI
*Bacillus* licheniforms	BaL	ATCC9859 /ATCC9789	Facultative anaerobe	37	8	BHI
*Bacillus* sphaericus	BaSph	ATCC4525 /LMG22257	Aerobic	37	7.0	NA
Lysinibacillus sphaericus	BaLy	BMZ008183	Aerobic	37	7.2	NA
*Bacillus* pasteurii	BaP	B80469 (from NCIM2477)	Aerobic	30	7.0	CASO+ 20 g/L urea

### 2.3 Identification of spores

The bacterial liquid of *bacillus* was undergone sterilization process through pasteurization, which involves heating to 80°C for 20 min followed by rapid cooling in ice water for 5 min. This step was crucial in reducing the number of vegetative cells present in the bacterial liquid, then the liquid was centrifuged (BIORIDGRE TGL-18M, Shanghai, China) at 7,000 rpm, 4°C for 7 min to separate and harvest the spores. A portion of these spores were stained for further analysis, which were placed on slides using malachite green dye and safranin counterstaining, subsequently, they were examined under confocal laser scanning microscope (Leica TCS-SP8, Germany) to observe their characteristics and morphology.

### 2.4 Preparation of dental self-healing resin composites

The collected spores from *Bacillus* and Bacterial liquid from non-Bacillus were washed, then centrifuged to remove excess liquid, resulting in a paste-like substance, which was subjected to freeze drying (BiLon, Bilang, Shanghai, China) for a period of 3 days, ultimately grinded to yield the lyophilized bacterial/spores.

The basic resin consisted of 49wt% BisGMA, 49wt% TEGDMA, 0.4wt% CQ, and 1.6wt% EDMAB, which is mixed magnetically at room temperature for 24 h, then combined with barium glass powder, nutrient powder (consisting of calcium lactate:yeast extract:glucose = 5:8:20) and bacterial powder (basic resin: barium glass powder: nutrient powder: bacterial powder = 45wt%:45wt%: 5wt%:5wt%) using a dual-center dispersion mixer known as the SpeedMixer (DAC150.1 FVZ-K, FlackTek, Inc., Germany) and a three-roll milling machine (EXAKT 80E, Exakt, Germany) for thorough homogenization.

### 2.5 Preparation of self-healing resin samples

**FIGURE 2:**
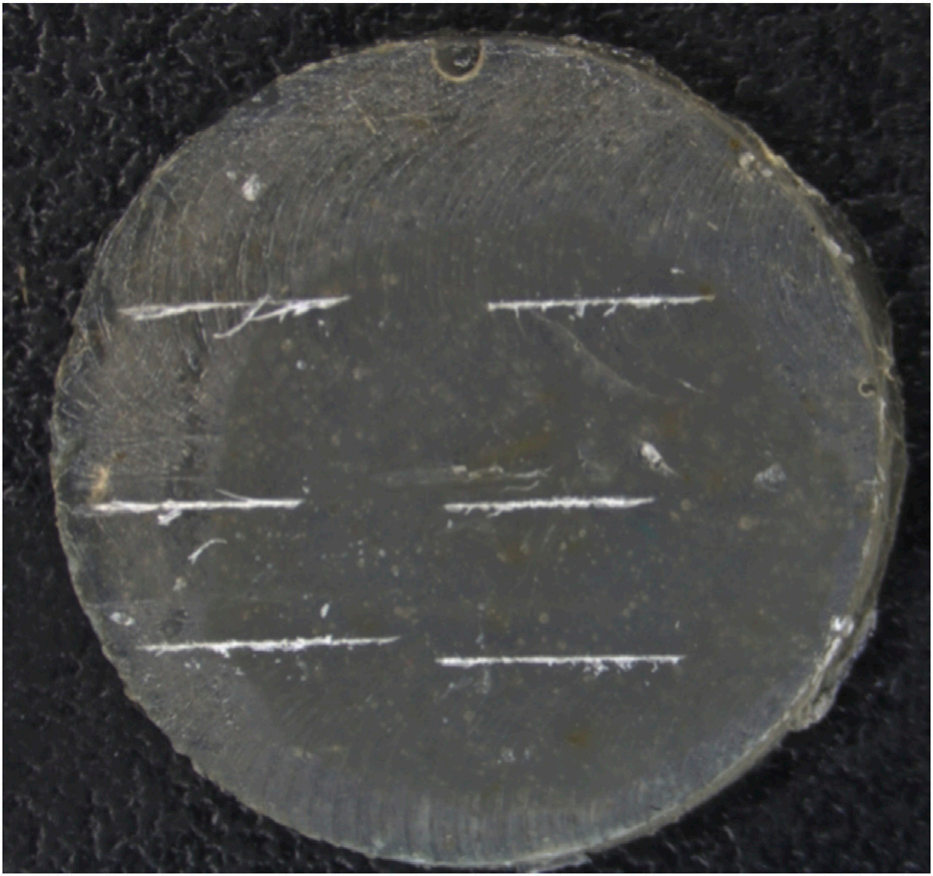
The resin disc samples with six artificial scratches.

Resin composites were injected into the silicone rubber mold of the disc and light-cured with a light-curing lamp (blue light, 470 nm, SLC-VIII B, Sifang, Hangzhou, China) for 60 s on each side. The samples were then polished with silicon carbide paper (P1500). Finally, they were formed into disc samples with a thickness of 1 mm (Φ = 10 mm), with two samples prepared for each group. After being placed for 1 day, a surgical blade was used to carve six artificial cracks of identical length (2 mm) onto the surface of each resin disc (Figure 2).

### 2.6 Evaluation of self-healing capabilities

The resin disks were placed in artificial saliva and stored at 37°C. They were photographed under a stereo-microscope on days 0, 1, 3, 5, 7, 14, 21, 30, 45, and 60. The area of the cracks was calculated using ImageJ image processing software, and differences at different time points were compared to obtain the initial self-healing data. On day 60, one resin disk sample was taken and dried overnight in an oven at 60°C. The morphology and microstructure of the healing surface were further observed using SEM (FE-SEM, S-4800, HITACHI, Japan). Another resin disk was re-carved along the original line, and the above steps were repeated to obtain the secondary self-healing data. The healing efficiency $\left(\eta 1\right)$ was calculated according to Formulas 1.

$$\eta 1=\frac{HA}{SA}\times 100\%$$ (1)

Where HA = healing area, SA = scratch area.

### 2.7 Statistical analysis

Statistical analysis was conducted using SPSS 27.0 software. Healing areas at different time were expressed as mean ± standard deviation. One-way analysis of variance (ANOVA) was employed for statistical comparisons, followed by the LSD method for datasets exhibiting homogeneous variance and Tamhane’s T2 method for those with heterogeneous variance. The significance level was set at p < 0.05 (*: p < 0.05, **: p < 0.01, ***: p < 0.001, ****: p < 0.0001).

## 3 Results

### 3.1 Characterization of bacteria/spores

**FIGURE 3:**
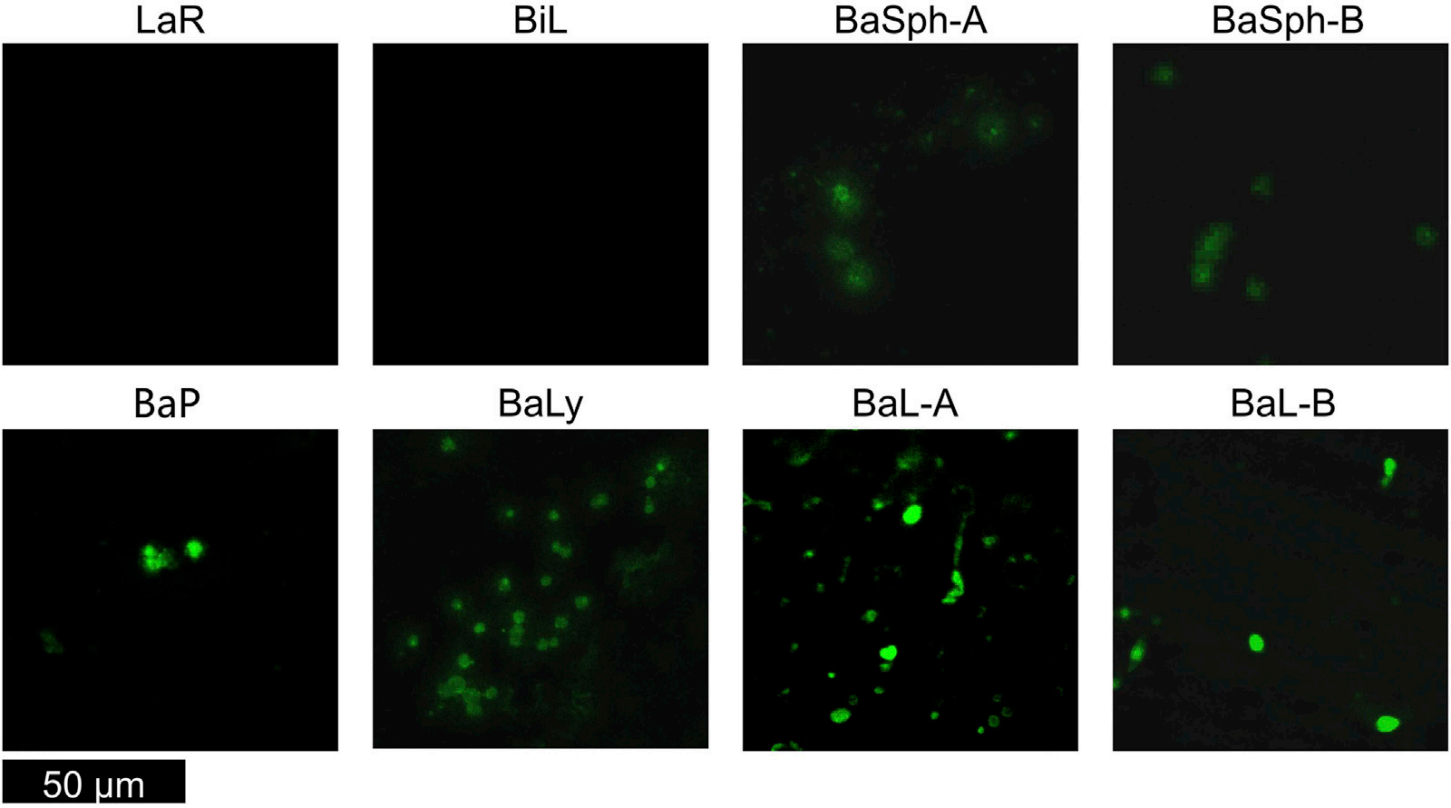
Confocal laser scanning microscope images of various non-spore-forming bacteria and *Bacillus* spores. Non-spore-forming bacteria was simplified by their absence of spores, the stained spores of *Bacillus* standed out as green.

Upon observation of the microscope photos, it is apparent that the stained spores standed out as green distinctly. The removal of red bacteria during centrifugation allowed a clear visualization of spores for *Bacillus*, different strains of which exhibited variability in size and shape, underscoring the diversity within this bacterial species. Identifying non-spore-forming bacteria was simplified by their absence of spores (Figure 3). It has also been demonstrated that the vegetative cells were effectively eradicated.

### 3.2 Initial self-healing process

**FIGURE 4:**
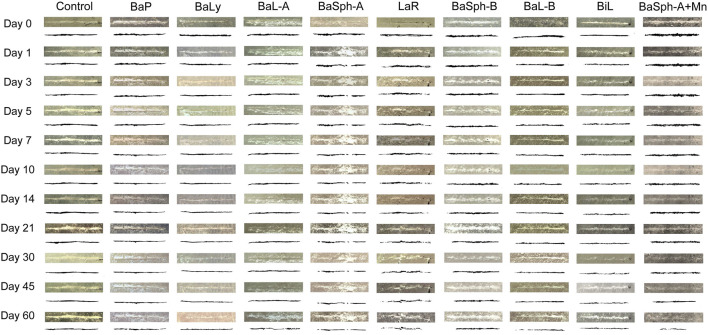
Observation and measurement of the initial self-healing of scratches of resin splines (0–60 days) under stereomicroscope (3.2x). The control group displayed minimal changes in scratches through 60 days, in contrast, the cracks in the other groups exhibited varying degrees of reduction and partial healing.

Figure 4 showed the progression of initial self-healing cracks in nine groups of dental self-healing resin discs and a control group, as observed under an *in vivo* stereomicroscope. The control group displayed minimal changes in scratches through 60 days, in contrast, the cracks in the other groups exhibited varying degrees of reduction and partial healing, with BaSph-A + Mn and BiL demonstrating the most significant reduction.

**FIGURE 5:**
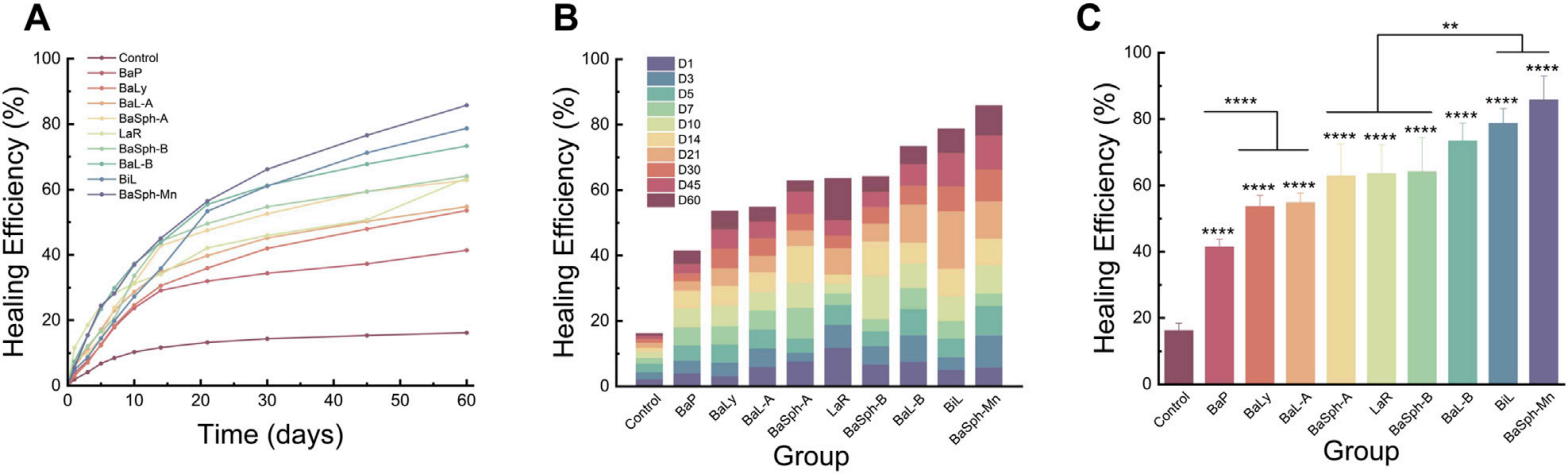
**(A)** Line chart and **(B)** stacked bar chart of comparation of initial-healing rate in different groups in 60 days. **(C)** Bar chart of comparation of initial-healing rate in different groups at day 60. *P < 0.05, **P < 0.01, ***P < 0.001, ****P < 0.0001.

Figure 5A presents a comparison of the healing status over the first 60 days among the nine groups of dental self-healing resin discs and the control group. Figure 5B depicts the cumulative healing rate of the nine groups of dental self-healing resin discs and the control group over the initial 60-day period. Furthermore, Figure 5C illustrates the healing rates on the 60th day of the first 60 days, highlighting the self-healing efficiency of BaP resin discs as the lowest at 41.4%, followed by BaLy at 53.6% and BaL-A at 54.8%, however, all three were significantly more effective than the control group at 16.2% (P < 0.00001); the highest healing efficiency was observed in the resin discs prepared with BaSph-A + Mn, BiL and BaL-B, all of which exceeded 70%. Among these three groups, the healing efficiency of BaSph-A + Mn was particularly impressive at 85.8%, although there was no significant difference between the three groups (P > 0.05). Following closely behind were BaSph-A, BaSph-B and LaR, all of which were effective in inducing CaCO_3_ precipitation with healing rates around 63%. The initial healing rates (mean ± standard deviation) of each group at different time points are shown in Table 2.

**TABLE 2:** Initial-healing rate (means±standard deviations, %) (n = 12) in different groups.

Time	Control	BaP	BaLy	BaL-A	BaSph-A	LaR	BaSph-B	BaL-B	BiL	BaSph-Mn
Day 0	0	0	0	0	0	0	0	0	0	0
Day 1	1.9 ± 0.8	3.8 ± 0.8	3.0 ± 0.6	5.8 ± 2.8	7.4 ± 4.3	11.6 ± 4.4	6.5 ± 4.6	7.4 ± 3.4	4.8 ± 4.1	5.6 ± 4.0
Day 3	4.2 ± 0.9	7.7 ± 2.0	7.1 ± 1.9	11.4 ± 3.7	10.1 ± 3.6	18.6 ± 4.0	12.1 ± 5.9	15.5 ± 4.1	8.7 ± 5.8	15.5 ± 1.0
Day 5	6.8 ± 1.3	12.4 ± 3.4	12.7 ± 2.8	17.2 ± 3.7	14.5 ± 4.7	24.7 ± 5.4	16.7 ± 6.7	23.4 ± 4.7	14.5 ± 6.7	24.4 ± 2.9
Day 7	8.5 ± 1.6	17.9 ± 4.3	18.3 ± 3.2	23.0 ± 2.8	23.9 ± 8.6	28.2 ± 5.8	20.4 ± 7.7	29.9 ± 5.4	19.8 ± 7.5	28.2 ± 3.8
Day 10	10.3 ± 2.0	23.8 ± 5.1	24.6 ± 3.0	28.7 ± 3.1	31.5 ± 8.1	31.3 ± 6.3	33.6 ± 8.2	37.3 ± 6.5	27.3 ± 9.7	37.0 ± 7.5
Day 14	11.7 ± 2.1	29.12 ± 5.5	30.6 ± 2.7	34.8 ± 3.8	42.8 ± 8.6	34.1 ± 6.7	44.2 ± 9.1	43.8 ± 6.5	35.8 ± 8.9	45.1 ± 9.2
Day 21	13.3 ± 2.1	32.0 ± 5.6	35.9 ± 2.9	39.8 ± 3.3	47.5 ± 8.5	42.1 ± 7.8	49.6 ± 12.6	55.5 ± 7.7	53.4 ± 4.9	56.4 ± 7.8
Day 30	14.4 ± 2.1	34.4 ± 5.2	42.0 ± 3.7	45.2 ± 3.0	52.6 ± 8.7	45.9 ± 6.2	54.8 ± 8.5	61.3 ± 5.3	61.1 ± 5.7	66.2 ± 10.1
Day 45	15.4 ± 2.2	37.3 ± 3.5	47.9 ± 3.5	50.3 ± 2.7	59.4 ± 8.7	50.7 ± 5.8	59.4 ± 8.4	67.7 ± 4.6	71.2 ± 7.4	76.6 ± 9.8
Day 60	16.2 ± 2.1	41.4 ± 2.2	53.6 ± 3.1	54.8 ± 2.8	62.8 ± 9.0	63.5 ± 8.2	64.1 ± 9.8	73.3 ± 5.2	78.7 ± 4.2	85.8 ± 6.5

**FIGURE 6:**
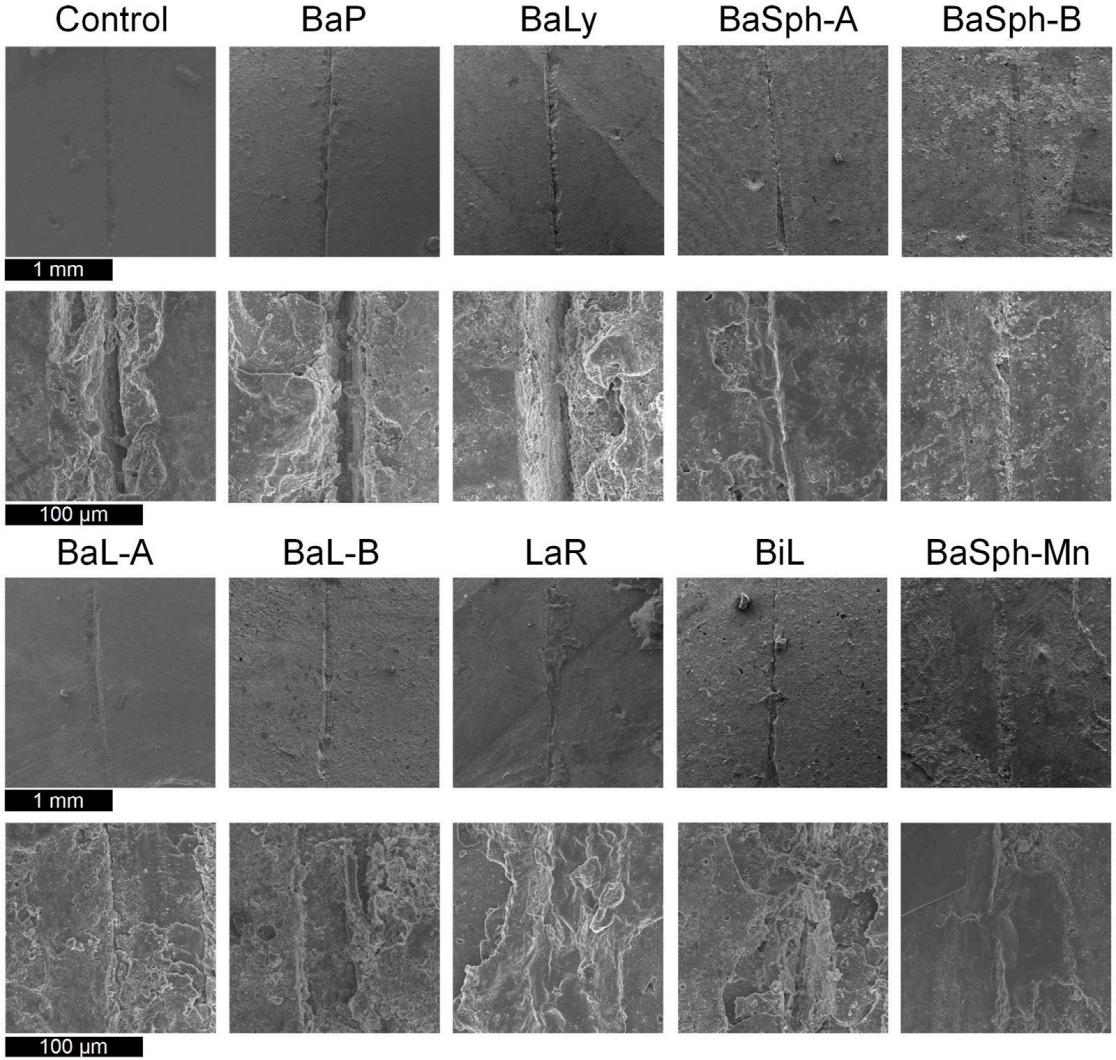
SEM images of the initial healing of cracks of resin samples (day 60), a white substance can be observed within the scratches of the bacterial groups.

Figure 6 offers a more detailed view of the healing process observed in the scratches through SEM images, the results aligned with the previous assessments of healing efficiency. In the magnified images, a white substance can be observed within the scratches of the bacterial groups, with BaSph-A + Mn exhibiting the most pronounced crack healing which can be attributed to the effective induction of spore formation by Mn^2+^, which significantly boosts CaCO_3_ production. Furthermore, BaSph-A, BaSph-B, BaL-A, BaL-B, LaR and BiL demonstrated varying degrees of healing, while the control group along with BaP and BaLy groups showed no significant healing effect.

### 3.3 Secondary self-healing process

**FIGURE 7:**
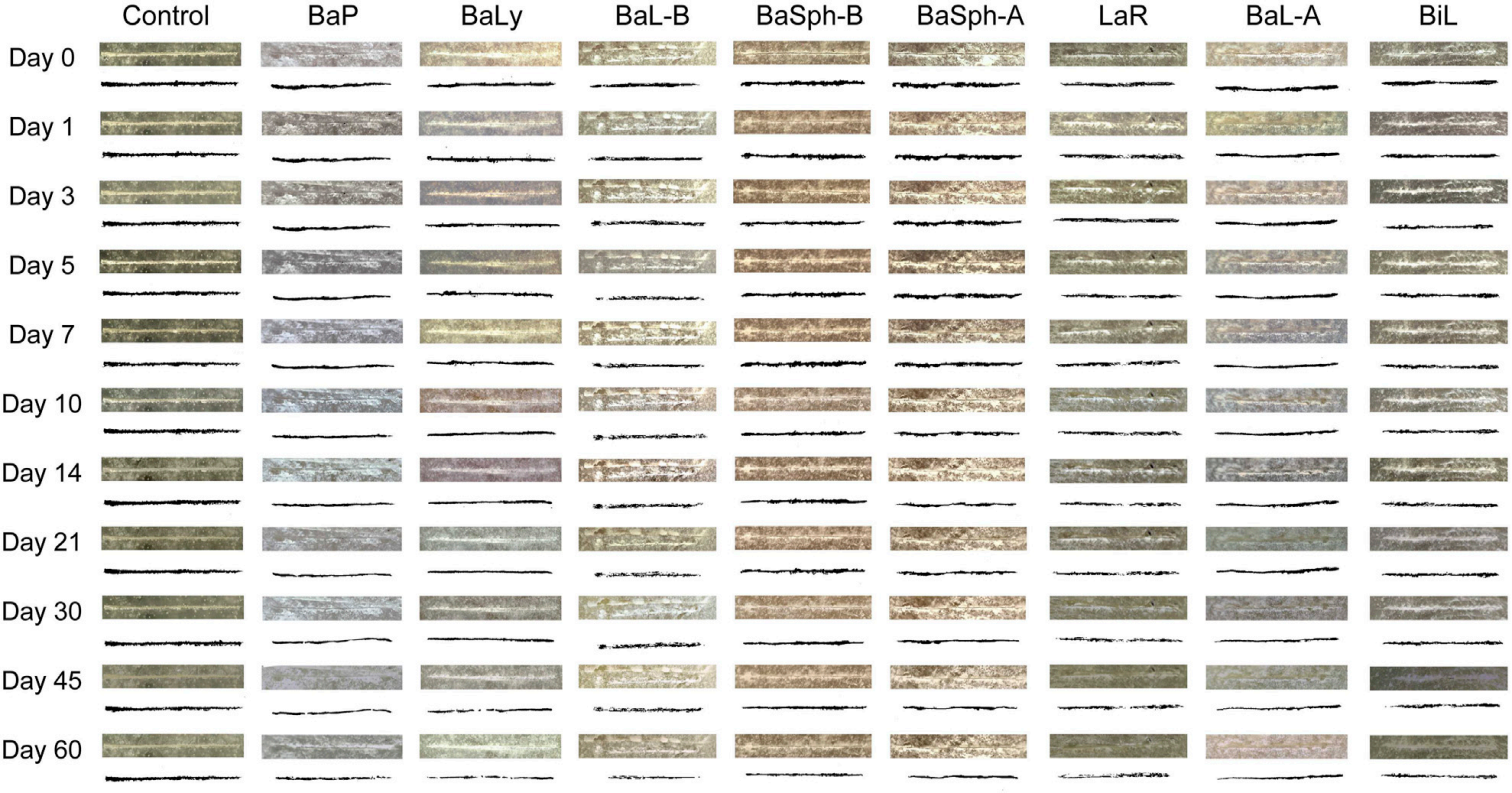
Observation and measurement of the secondary self-healing of scratches of resin splines (0–60 days) under stereomicroscope (3.2x). The control group’s scratches remained relatively unchanged from day 1 to day 60, while the experimental groups displayed varying degrees of shallow, resembling the initial healing situations.

Figure 7 compared the healing progress of resin discs in eight different groups of dental self-healing resin discs and the control group over repeated healing experiment. The results indicated that the control group’s scratches remained relatively unchanged from day 1 to day 60, while the experimental groups displayed varying degrees of shallow, resembling the initial healing situations.

**FIGURE 8:**
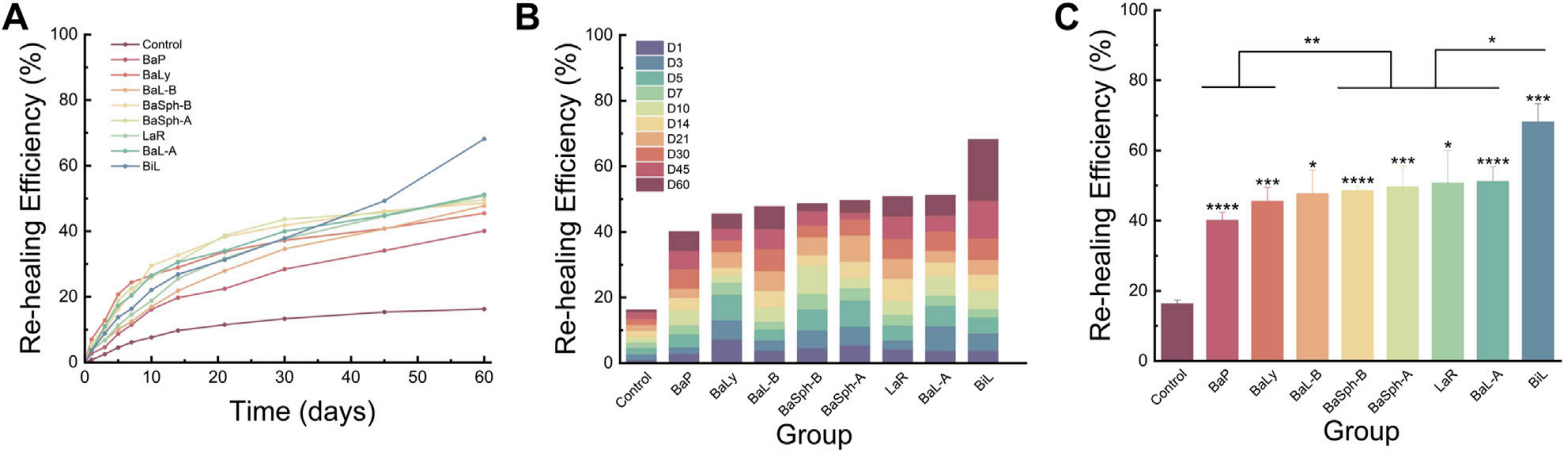
**(A)** Line chart and **(B)** stacked bar chart of comparation of secondary-healing rate in different groups in 60 days. **(C)** Bar chart of comparation of secondary-healing rate in different groups at day 60. *P < 0.05, **P < 0.01, ***P < 0.001, ****P < 0.0001.

Figures 8A,B provided representation of the healing process for the eight groups of dental self-healing resin discs and the control group. Additionally, Figure 8C highlights the healing progress on day 60, revealing that BaP had the lowest self-healing efficiency at 40.1%, consistent with the initial healing results. The BaLy group followed closely behind with an efficiency of 45.5%, both significantly better than the control group’s efficiency of 16.2% (P < 0.001). The resin disc with BiL was demonstrated with the highest healing efficiency at 68.2%, while the remaining experimental groups showed similar healing rates around 50%, indicating effective promotion of CaCO_3_ precipitation. The secondary healing rates (mean ± standard deviation) of each group at different time points are shown in Table 3.

**TABLE 3:** Secondary-healing rate (means±standard deviations, %) (n = 6) in different groups.

Time	Control	BaP	BaLy	BaL-B	BaSph-B	BaSph-A	LaR	BaL-A	BiL
Day 0	0	0	0	0	0	0	0	0	0
Day 1	0.8 ± 0.5	2.7 ± 0.4	7.0 ± 5.7	3.6 ± 0.7	4.3 ± 0.2	5.3 ± 2.2	4.0 ± 1.5	3.6 ± 1.3	3.6 ± 1.0
Day 3	2.5 ± 1.0	4.7 ± 1.5	12.9 ± 7.4	6.8 ± 0.7	9.8 ± 0.4	10.9 ± 2.9	6.8 ± 2.0	11.1 ± 3.5	8.9 ± 1.6
Day 5	4.5 ± 1.0	8.7 ± 3.5	20.8 ± 10.3	10.1 ± 1.7	16.2 ± 1.0	18.9 ± 4.1	11.3 ± 1.8	17.3 ± 2.8	13.7 ± 1.6
Day 7	6.1 ± 1.8	11.4 ± 4.9	24.4 ± 9.3	12.5 ± 1.8	20.9 ± 0.6	22.7 ± 2.6	14.6 ± 1.5	20.4 ± 2.7	16.4 ± 2.9
Day 10	7.7 ± 1.3	16.2 ± 5.6	26.6 ± 8.6	17.0 ± 2.3	29.5 ± 1.2	25.8 ± 4.4	18.8 ± 2.2	26.5 ± 6.6	22.1 ± 2.8
Day 14	9.7 ± 1.3	19.7 ± 4.9	28.9 ± 8.4	21.8 ± 3.6	32.7 ± 1.2	30.8 ± 4.2	25.6 ± 4.4	30.6 ± 5.8	26.9 ± 2.3
Day 21	11.5 ± 2.1	22.5 ± 5.1	33.7 ± 7.9	27.9 ± 4.0	38.3 ± 1.4	38.7 ± 3.9	31.7 ± 5.5	34.0 ± 4.4	31.3 ± 3.6
Day 30	13.3 ± 2.0	28.4 ± 4.7	37.2 ± 7.0	34.6 ± 5.6	41.8 ± 1.9	43.7 ± 3.9	37.7 ± 7.1	40.0 ± 5.1	37.8 ± 1.7
Day 45	15.4 ± 1.2	34.1 ± 3.2	40.8 ± 6.0	40.7 ± 6.1	46.2 ± 1.0	45.7 ± 3.8	44.6 ± 8.5	44.8 ± 5.2	49.3 ± 5.6
Day 60	16.3 ± 1.0	40.1 ± 2.1	45.5 ± 3.6	47.7 ± 6.0	48.6 ± 1.1	49.6 ± 4.8	50.8 ± 8.3	51.2 ± 3.8	68.2 ± 4.6

**FIGURE 9:**
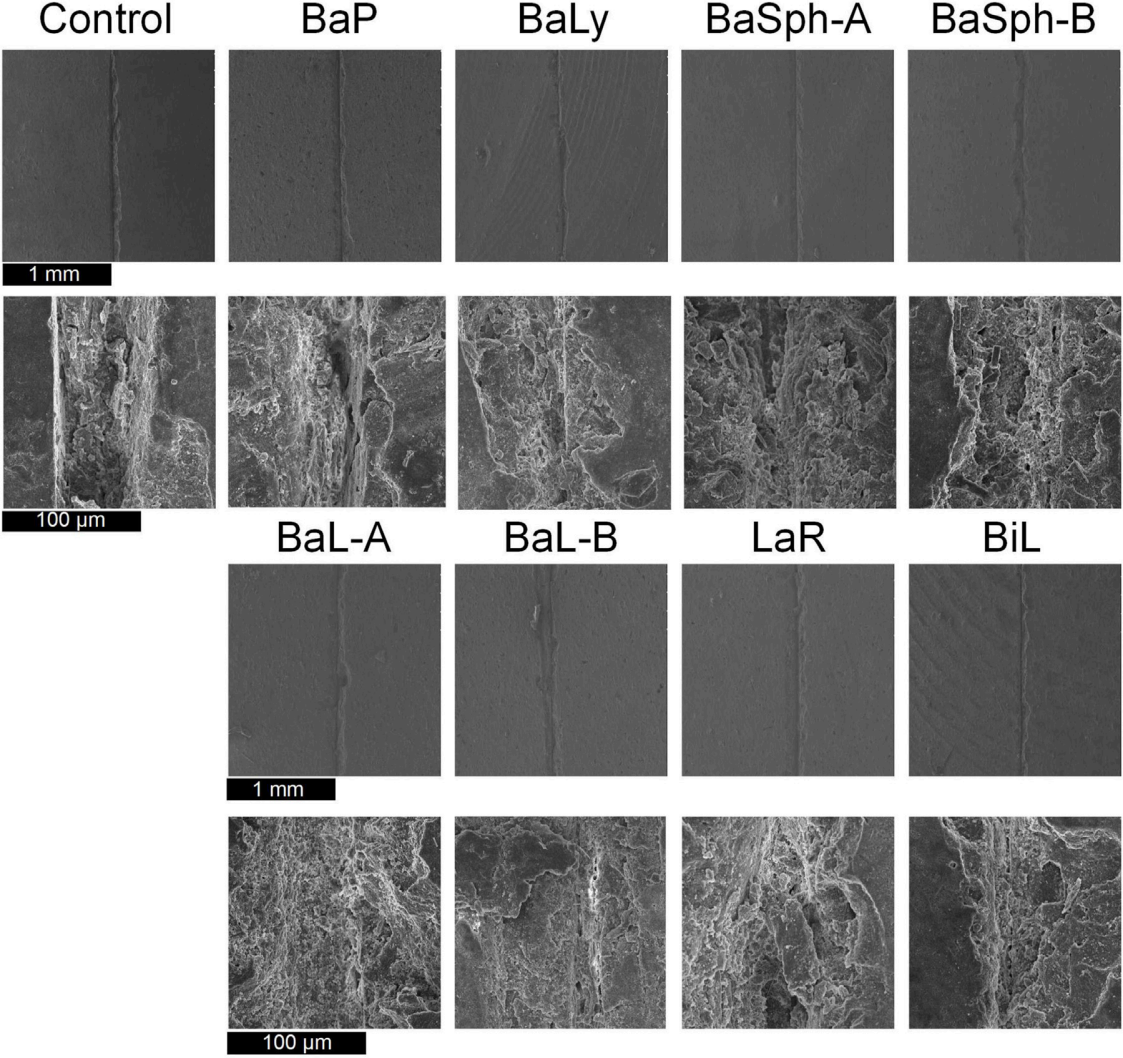
SEM images of the secondary healing of cracks of resin samples (day 60), a white substance can be observed within the scratches of the bacterial groups.

Furthermore, SEM images of the secondary healing phase (Figure 9) supported these findings, showing varying degrees of healing in the BaL-A, BaL-B, BaLy, BaSph-A, BaSph-B, BiL and LaR groups, while the control and BaP groups displayed less significant improvements.

### 3.4 Comparative analysis of two self-healing processes

**FIGURE 10:**
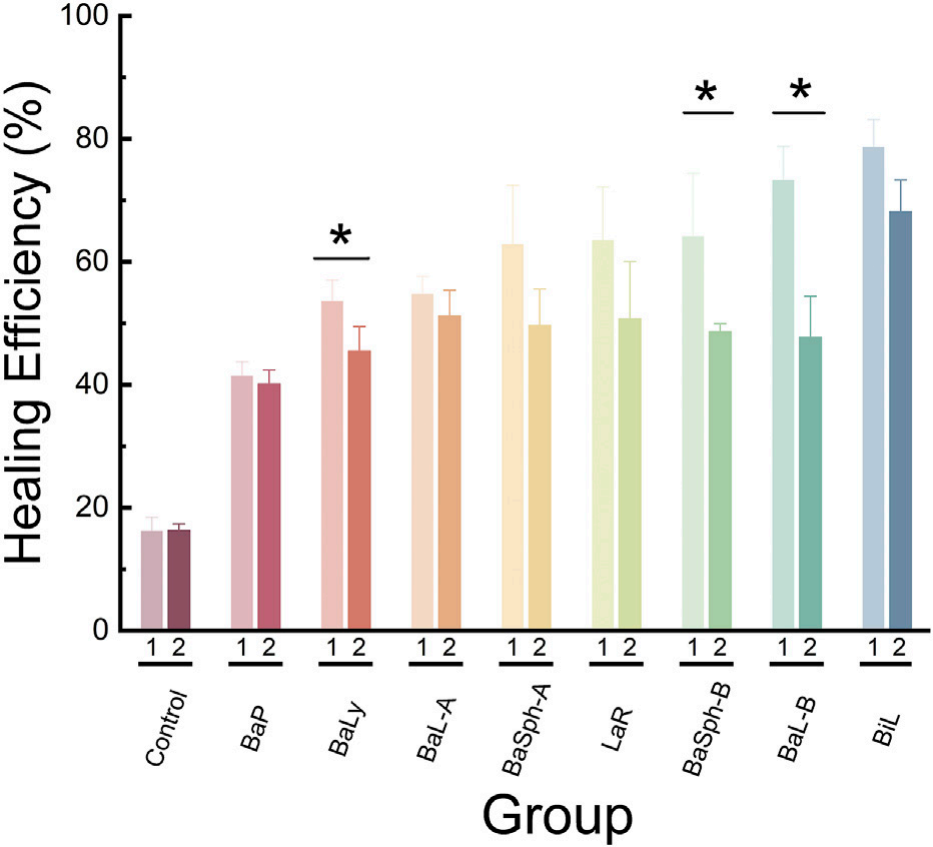
Comparation of healing rate and re-healing rate in different groups at day 60. *P < 0.05, **P < 0.01, ***P < 0.001, ****P < 0.0001.

Figure 10 illustrated a comparison of the healing rates of different strains of bacteria during initial and secondary healing. It is evident that the control group had the lowest healing rate during secondary healing and showed no significant difference compared with initial healing group. The healing rates of other strains of bacteria during secondary healing were lower compared to initial healing groups, with strains BaP, BiL, BaL-A and BaSph-A showing no significant difference (P > 0.05). The secondary healing rates of BaL-B, BaSph-B and BaLy significantly decreased (P < 0.05), but still maintain a self-healing efficiency of around 50%. This decrease in healing efficiency may be attributed to a slight loss of nutrients, suggesting the need to consider increasing the proportion of nutrients in future studies.

In conclusion, the most effective strain for inducing CaCO_3_ precipitation and displaying significant self-healing abilities is BiL, followed by BaL-B, BaSph-A, BaSph-B, BaL-A and LaR exhibit comparable levels of self-healing efficiency. BaP and BaLy, on the other hand, show less effectiveness in self-healing. The inclusion of Mn^2+^ has been found to enhance spore yield and improve self-healing capability.

## 4 Discussion

Over time, microcracks gradually develop in resin restorations, eventually causing fractures. Previous studies on self-healing dental resin composites have focused mainly on re-bonding close to complete fracture. However, in the oral environment, resin restorations are constantly subjected to biting forces, making it impractical for them to re-bond statically after fracturing. In fact, cracks and excessive pressure can lead to the direct fracture and detachment of dental restorations at any time. Therefore, assessing self-healing properties solely based on re-bonding close to complete fracture is inaccurate. Microcracks typically form before complete fracture, and if they can be continuously self-healed, the likelihood of resin fracture would be significantly reduced. Early self-healing of microcracks can prevent further damage. Hence, prioritizing the evaluation of early self-healing performance is essential.

Given the continuous use of restorations, the ability of self-healing materials to repair themselves repeatedly is crucial. MICP was emerged as an innovative approach for repeated self-healing of microcracks in dental resin composites. This study investigated the initial and secondary self-healing processes of cracks in resin restorations under simulated oral conditions, in which the area changes of microcracks were observed and quantitatively analyzed a microscopic level, providing a more accurate evaluation of the initial and secondary self-healing efficiencies of self-healing resin composites of eight bacterial stain groups, covering the majority of the bacterial species studied in self-healing concrete.

In our study, each group consisted of either 6 or 12 samples. This sample size was determined by aligning with common practices in the field, considering the effect size and variability, and taking into account resource constraints. However, the relatively small sample size may limit the generalizability of our findings. Future research will explore larger sample sizes and more advanced techniques. Additionally, the 60-day observation period was selected because prior research on self-healing concrete has varied significantly in duration, with many studies spanning only 30 days. Given that bacterial-induced calcium carbonate formation generally requires a longer timeframe, we extended the observation period to 60 days to obtain more comprehensive and reliable data. Moreover, the present quantitative method employing stereomicroscopy for image acquisition exhibits certain limitations, primarily due to the involvement of subjective judgment. Future research endeavors will focus on exploring more objective techniques and tools to enhance the quantification of self-healing effects. Lastly, our study did not pursue qualitative analysis of the substance, as fundamental analytical tools such as EDS cannot provide a definitive confirmation, which is partly due to the semi-quantitative nature of EDS and the fact that our filler composition also contains Ca^2+^, while the substance in question has been consistently identified as CaCO_3_ in prior investigations of self-healing concrete (Wang J.-Y. et al., 2012; Wang J. et al., 2012; Achal et al., 2013; Wang et al., 2014a; Wang et al., 2014b; Wang et al., 2014c). Certainly, some studies have mentioned that X-ray Diffraction (XRD) (Wang J.-Y. et al., 2012; Wang J. et al., 2012) and Fourier Transform Infrared Spectroscopy (FTIR) (Achal et al., 2013) can be used to analyze crystalline materials and confirm the formation of calcium carbonate. We will address this gap in our future research by providing the relevant data.

The microorganisms and nutrients employed in this study are essential active ingredients in self-healing materials. *Bacillus* sphaericus and *Bacillus* licheniformis demonstrated high calcium carbonate production in specific condition (Seifan et al., 2016b), and self-healing concrete containing these bacteria, calcium lactate, and yeast extract can effectively repair cracks up to 0.46 mm in width (Wiktor and Jonkers, 2011). In this study, spores of *bacillus* were incorporated into resin composites instead of bacteria in the previous study (Seifan et al., 2020). The polymerization shrinkage of dental resin composites and changes in the oral environment can hinder bacterial biomineralization. However, *Bacillus* licheniformis, known for its resilient spores, can survive in challenging conditions, it is a promising candidate for dental materials, especially in the oral cavity’s fluctuating temperatures.

Using a protective carrier to immobilize bacteria could offer a more effective solution, but additional research is required to further explore this possibility. Furthermore, this study demonstrated that the addition of Mn^2+^ to *Bacillus* culture significantly improved its self-healing abilities, resulting in superior performance compared to other strains, which is attributed to the ability of Mn^2+^ to enhance spore production per unit time (Xu et al., 2018). Therefore, future research efforts could concentrate on selected strains that effectively induce CaCO_3_ precipitation and incorporating Mn^2+^ into *Bacillus* incubation to enhance self-healing properties.

Since Metchnikoff’s pioneering work in 1907 (Metchnikoff, 2004), the definition of probiotics has significantly evolved. They are now defined as safe, beneficial, living microorganisms, primarily bacteria, that improve human health (Fuller, 1989) by regulating intestinal flora and enhancing immune function (Suez et al., 2019). Recent studies also highlight their potential benefits in oral health, including reducing harmful bacteria and preventing dental caries, periapical inflammation, and periodontal disease (Kumar et al., 2021; Homayouni Rad et al., 2023; Shi et al., 2023; Luo et al., 2024). The findings of this study highlighted the significant potential of Bifidobacterium longum and *Bacillus* licheniforms as powerful microorganisms with inherent self-healing properties, which were consistent with the results of previous study (Seifan et al., 2020), meanwhile, they are valuable probiotics which have been shown to be benign to the human body, which can actually assist in maintaining a healthy balance within both the gastrointestinal and oral microbiomes when incorporated into dental materials. Meanwhile, *Bacillus pasteurella* (B80469) had the weakest self-healing effect in the study, it referred that it may due to the inappropriate temperature of the oral environment, because *Bacillus pasteurella* should be cultivated at 30°C.

In addition to microorganisms, essential nutrients for their survival are crucial. Previous study (Seifan et al., 2020) on self-healing dental composites used urea (which can raise oral pH, harming cells and flora) and calcium chloride (which releases heat, risking burns and absorbing into the mucosa), which have toxic effects on organs and can corrode oral prostheses (Seifan et al., 2016a). Thus, they are unsuitable for dental materials. In contrast, this study used calcium lactate and yeast extract, which are common in oral care products. Calcium lactate forms CaCO_3_ to heal dental resin composites safely, while yeast extract nourishes oral cells and maintains microbiota balance. However, it is important to investigate if dietary nutrients like calcium and glucose can compensate for oral nutrient losses. Moreover, microbial self-healing agents with poor compatibility can weaken dental resin composites. Future goals include using biocompatible and mechanically superior alternatives like polylactic acid. The study found that nutrient content decreases over time, reducing self-healing efficacy. The samples in this study were immersed in an artificial saliva solution composed of NaCl, KCl, CaCl_2_, NaH_2_PO_4_, urea and Na_2_S, which mimics the composition and concentration of human saliva, thereby simulating the compensation of nutrients. While higher nutrient concentrations improve healing, they may increase costs and compromise mechanical properties. The proportion of the self-healing component in this study was set at 5% based on prior research (Huyang et al., 2016; Yahyazadehfar et al., 2018), which ensures high self-healing efficacy without compromising mechanical properties. However, determining the optimal dosage and ratio of probiotics and nutrients is vital for cost management and minimizing loss. In this study, microorganisms and nutrients were meticulously integrated into the fundamental components of the dental resin, this methodological approach aligns with the current best practices within the dental materials community, thereby ensuring both safety and efficacy. A series of comprehensive compatibility assessments will be conducted to ensure that this integration does not conflict with existing materials.

Moreover, resin composites commonly face issues with developing secondary caries due to polymerization shrinkage. Through the utilization of microbial self-healing technique, bacteria present within resin restoration may be activated during polymerization shrinkage and gap formation, which triggers biomineralization, leading to the creation of CaCO_3_ or hydroxyapatite (HAP), thereby reducing the occurrence of secondary caries. Future research will focus on determining the effectiveness of this innovative self-healing dental resin composite that relies on biomineralization, in repairing microcracks and preventing secondary caries simultaneously, which aims to improve the overall success rate of resin restorations. Additionally, in environments rich in phosphates, CaCO_3_ has the potential to convert into HAP (Wang et al., 2021). Further exploration may involve investigating ways to facilitate the conversion of CaCO_3_ surrounding cracks into HAP, which would contribute to the enhanced restoration of tooth tissue and reinforcement of the mechanical properties of resin restorations.

## 5 Conclusion

This study is dedicated to the utilization of bacteria-mediated biomineralization technique in developing an innovative, biocompatible dental self-healing resin composite, capable of autonomously detecting and repairing cracks in simulated oral environments, which has demonstrated remarkable long-term self-healing abilities, including the capacity to repair the same crack twice. The bacteria, including beneficial probiotics, such as B81617 Bifidobacterium longum and ATCC9789 *Bacillus* licheniformis were selected based on their exceptional repeated self-healing properties. These strains have consistently demonstrated a remarkable healing efficiency that exceeds 70%, positioning them as exceptionally effective candidates for applications where consistent and reliable self-healing properties are essential. Meanwhile, the self-healing effect of *Bacillus* was significantly enhanced by the addition of Mn^2+^ during sporulation culture, which in turn increased the spore yield. These findings represent a significant advancement in the field of dental self-healing resin composites and offer a promising solution to the widespread issue of resin restoration fractures.

## Data Availability

The original contributions presented in the study are included in the article/supplementary material, further inquiries can be directed to the corresponding authors.
